# Sex differences in underweight and body mass index in Chinese early de novo patients with Parkinson's disease

**DOI:** 10.1002/brb3.1893

**Published:** 2020-10-16

**Authors:** Qing Wu, Meizhen Liu, Ming Yu, Jianfei Fu

**Affiliations:** ^1^ Department of Neurology Ningbo First Hospital Ningbo ZheJiang China; ^2^ Department of Neurology Ningbo Municipal Hospital of TCM Ningbo ZheJiang China; ^3^ Department of Medical Record Ningbo First Hospital Ningbo ZheJiang China

**Keywords:** body mass index, Chinese, de novo, Parkinson's disease, sex differences, underweight

## Abstract

**Objective:**

There have been studies investigating sex differences in clinical manifestation of Parkinson's disease (PD). However, sex differences in underweight and body mass index (BMI) in de novo PD patients lacked systematic study. We aimed to compare sex differences in clinical features and related factors of underweight and BMI in Chinese de novo PD patients.

**Materials and Methods:**

A total of 253 untreated PD inpatients and 218 controls were recruited from Ningbo. BMI, demographics, Montreal Cognitive Assessment (MoCA), supine and upright blood pressure, Hamilton Anxiety Scale (HAMA), Hamilton Depression Scale (HAMD), homocysteine (HCY), uric acid, glycated hemoglobin, and lipid parameters were examined. Patients were assessed using the Unified Parkinson's Disease Rating Scale (UPDRS) motor scores and Hoehn and Yahr (HY) Rating Scale.

**Results:**

Female patients had a significantly lower incidence of underweight and higher BMI than male patients, and there were sex differences in serum lipids, HCY levels, and depression severity. Binary regression analysis showed that only in male patients was underweight associated with the UPDRS motor score and lower ΔSBP and ΔDBP values (all *p* < .05). Further multiple regression analysis indicated, in addition to the correlations between BMI and ΔSBP and ΔDBP values in both sexes (all *p* < .001), BMI was also associated with MoCA and lower UPDRS motor scores in male patients and lower HAMD scores in female patients.

**Conclusion:**

Our study suggests that there are significant sex differences in the prevalence of underweight, BMI, and factors associated with underweight and BMI among de novo PD patients.

## Graphical abstract

Sex differences in underweight and body mass index in Chinese early de novo patients with Parkinson's disease (PD) have not been investigated before, our study suggests that there are significant sex differences in the prevalence of underweight, body mass index (BMI), and factors associated with underweight and BMI among de novo PD patients; therefore, sex‐specific management strategies such as dietary structure, physical activity, and nutritional status interventions may have certain clinical significance for improving life quality and prognosis of patients.

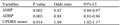

## 1 INTRODUCTION

The motor and nonmotor symptoms of Parkinson's disease (PD) have been widely explored; however, there are limited studies on sex differences in clinical features, and the results are inconclusive. A study of early PD patients showed that arm, face, and neck symptoms were more severe in men and postural disorders were more pronounced in women. Among the nonmotor symptoms, compared with women, men had higher prevalence of REM behavior disorder (RBD) and orthostatic hypotension, and more severe cognitive impairment and sexual dysfunction (Szewczyk‐Krolikowski et al., 2014), whereas a Chinese study involving 428 early de novo PD patients revealed no significant sex differences in motor symptoms and showed that female patients were prone to be depressed and showed worse performance on cognitive function in the context of nonmotor symptoms (Song et al., 2014); many factors, including genetic susceptibility, sociocultural factors, and the neuroprotective effect of estrogen, may play a part in these differences (Picillo et al., 2013).

Evidence has shown that prominent weight loss occurs with the progression of PD, but there are few reports on sex differences in the prevalence of underweight, body mass index (BMI), and related factors. A Japanese study suggested that male PD patients had higher BMI levels than female PD patients (Nakamura et al., 2017). Another study revealed significant sex differences in the association between reduced BMI and depression severity among the PD population (Pilhatsch et al., 2013). Since some of the anti‐Parkinson drugs can also affect the symptoms (Erro et al., 2013; Honig et al., 2009), it is meaningful to investigate the sex differences in the natural characteristics of PD. Furthermore, sex differences in underweight and BMI in Chinese early de novo PD patients remain unknown; therefore, the objective of our study was to explore the sex differences in (a) the prevalence of underweight, BMI, demographic, and clinical features; and (b) the associated factors of underweight and BMI in newly diagnosed and drug‐naïve Chinese PD patients.

## 2 MATERIALS AND METHODS

### 2.1 Subjects

All subjects were recruited from Ningbo First Hospital, including 253 newly diagnosed PD inpatients (males/females = 146/107) from the Department of Neurology and 218 healthy people (males/females = 110/108) from the Physical Examination Center. None of these patients had taken anti‐Parkinson medications before. The study protocol was approved by the Clinical Research Ethics Committee of Ningbo First Hospital; a neurologist explained the basic procedures to subjects with appropriate language, and consent to participate in the study was obtained.

Parkinson's disease was diagnosed on the basis of the diagnostic criteria of the Movement Disorders Society (MDS; Postuma et al., 2015; because of the drug‐naïve patients in this study, the patients fulfilled the criteria of clinically probable PD), and patients who met the following criteria were excluded: (a) significant secondary peripheral neuropathy; (b) obvious cognitive impairment and inability to cooperate with the scale assessment; (c) illnesses that affect normal dietary intake (e.g., oral, throat, and digestive tract diseases); and (d) use of vasopressor or antihypertensive drugs.

### 2.2 General information and clinical measurements

Body mass index and demographics were collected, and the following three scales were assessed for all subjects by our neurologists: cognitive function was evaluated by the Montreal Cognitive Assessment (MoCA; Nasreddine et al., 2005); anxiety was evaluated by the Hamilton Anxiety Scale (HAMA; Hamilton, 1959); and depression was evaluated by the Hamilton Depression Scale (HAMD; Hamilton, 1960). Height and weight data were collected, and BMI was calculated (weight (kg)/[height (m)]^2^). Height was measured with the participant barefoot standing upright with a value accurate to 0.1 cm, and body weight was measured by an electronic weight scale with a value accurate to 0.1 kg. According to the Chinese Working Group (Li et al., 2010), the cutoff point for underweight was 18.5 for BMI in China, and all subjects were classified as underweight if their BMI was <18.5 and nonunderweight if their BMI was ≥18.5.

Then, subjects rested in the supine position for at least 5 min, and the systolic and diastolic blood pressure (SBP and DBP) of the brachial artery on the right arm were measured by sphygmomanometer (OMRON HEM‐7111, China). SBP and DBP at 1, 3, and 10 min after standing were also obtained, and the maximum change in SBP and DBP (ΔSBP, ΔDBP) was recorded. If subjects reported presyncope symptoms (e.g., nausea, dizziness) after switching to orthostatism, they needed to lie down immediately until the discomfort disappeared. In addition, the Hoehn and Yahr (HY) Rating Scale, the Unified Parkinson's Disease Rating Scale (UPDRS) motor scores, and the duration of PD were evaluated in patients.

### 2.3 Blood sample collection

Blood samples of all subjects were obtained between 7 and 9 a.m. after overnight fasting, and the sera were centrifuged at 1000 g for at least 5 min. Glycated hemoglobin (HbA1c), homocysteine (HCY), uric acid (UA), total cholesterol (TC), and low‐density lipoprotein (LDL), high‐density lipoprotein (HDL), and triglyceride (TG) levels were measured by using urate peroxidase coupling and direct methods in the clinical laboratory of Ningbo First Hospital.

### 2.4 Statistical analysis

Comparisons of demographic and clinical parameters between different groups were performed by using an independent‐sample *t* test or Mann–Whitney *U* test for continuous variables (the one‐sample Kolmogorov–Smirnov test was used to assess normality) and the chi‐square test for categorical variables. The comparison of the prevalence of underweight between male and female patients was performed by the chi‐square test, and further multiple logistic regression was conducted to correct for confounding factors. The related factors of underweight were analyzed by binary logistic regression in both sexes. The relationships between BMI and all other variables were determined by Pearson's or Spearman's correlation coefficient, as appropriate. The Bonferroni corrections were also applied to adjust for multiple testing. Further stepwise multiple regression analyses were used to identify significant predictive variables related to BMI in male and female patients. All statistical analyses were performed using SPSS version 21.0, and the significance level was set at a 2‐tailed *p* value of .05.

## 3 RESULTS

### 3.1 Baseline results

Table 1 shows that there was no significant difference in age and education years among the subgroups, female patients had higher TC, TG, and LDL‐C levels and HAMD values, and a lower HCY level and smoking prevalence than male patients (all *p* < .05). Compared with male controls, male patients had lower ΔSBP and ΔDBP values and HbA1c, TC, and LDL‐C levels and worse performance in cognitive function, anxiety, and depression (all *p* < .05). Regarding the female groups, PD patients have higher HCY and TC levels, have lower ΔSBP and ΔDBP values, and have more severe cognitive impairment, anxiety, and depression than female controls (all *p* < .05). There were no significant differences between male and female controls except in UA (*p* < .001), HCY (*p* < .001) levels, and the percentage of smokers (*p* < .001).

**TABLE 1:** Sex differences of demographic and clinical features between PD patients and healthy controls

	Male patients (*N* = 146)	Male control (*N* = 110)	Female patients (*N* = 107)	Female control (*N* = 108)	*p* Value^a^	*p* Value^b^	*p* Value^c^	*p* Value^d^
Age (years)	63.8 ± 10.7	65.5 ± 11.1	62.0 ± 9.9	64.8 ± 11.7	.22	.11	.17	.85
Education (years)	9.6 ± 2.3	9.8 ± 2.8	10.1 ± 2.6	10.5 ± 2.0	.56	.46	.27	.28
Current smokers	80 (54.8%)	61 (55.5%)	1 (0.9%)	1 (0.9%)	.91	.98	<.001	<.001
Former smokers	2 (1.4%)	3 (2.7%)	0 (0.0%)	0 (0.0%)	.43	—	.51	.25
Never smokers	64 (43.8%)	46 (41.8%)	106 (99.1%)	107 (99.1%)	.75	.98	<.001	<.001
Duration of disease (months)	18.2 ± 14.1		17.9 ± 13.8				.89	
HY	2.6 ± 0.9		2.5 ± 0.8				.36	
UPDRS motor	18.9 ± 9.2		17.2 ± 7.6				.13	
TC (mmol/L)	3.9 ± 1.1	4.2 ± 1.1	4.6 ± 1.2	4.1 ± 1.1	.03	.02	<.001	.92
TG (mmol/L)	0.8 ± 0.3	1.0 ± 0.2	1.2 ± 0.4	1.1 ± 0.3	.08	.26	.006	.13
HDL (mmol/L)	1.2 ± 0.6	1.3 ± 0.3	1.1 ± 0.4	1.3 ± 0.6	.41	.35	.29	.31
LDL (mmol/L)	2.4 ± 0.8	2.7 ± 0.8	2.9 ± 0.9	2.7 ± 0.9	.005	.27	<.001	.66
HbA1c (%)	4.8 ± 0.7	5.3 ± 1.3	4.8 ± 0.9	5.2 ± 1.2	.04	.08	.32	.07
HCY (µmol/L)	15.4 ± 12.9	13.6 ± 7.9	12.6 ± 8.1	10.4 ± 3.2	.19	.02	<.001	<.001
UA (µmol/L)	303.8 ± 112.5	337.1 ± 81.3	293.0 ± 93.1	295.8 ± 88.7	.07	.08	.12	<.001
MoCA	23.1 ± 4.5	27.4 ± 2.7	24.1 ± 4.9	26.6 ± 2.9	<.001	<.001	.08	.06
Visuospatial/executive	3.7 ± 0.9	4.9 ± 0.2	4.0 ± 0.9	4.7 ± 0.3	<.001	.03	.12	.24
Naming	2.1 ± 0.4	2.3 ± 0.5	2.1 ± 0.6	2.5 ± 0.4	.13	.22	.88	.35
Attention	4.6 ± 1.1	5.4 ± 0.8	4.8 ± 0.7	5.3 ± 0.6	.006	.02	.46	.66
Language	2.1 ± 0.8	2.4 ± 0.6	1.8 ± 0.7	2.6 ± 0.2	.03	.02	.04	.26
Abstraction	1.3 ± 0.5	1.8 ± 0.5	1.5 ± 0.5	1.9 ± 0.3	<.001	.008	.34	.71
Delayed recall	4.6 ± 0.8	4.7 ± 0.6	4.6 ± 0.7	4.6 ± 0.4	.25	.51	.75	.46
Orientation	5.1 ± 1.2	5.7 ± 0.7	5.3 ± 0.3	5.5 ± 0.3	.08	.13	.36	.28
HAMA	9.4 ± 4.3	6.5 ± 4.7	8.9 ± 4.5	7.2 ± 4.6	<.001	.003	.35	.33
HAMD	11.7 ± 6.4	5.9 ± 3.3	13.8 ± 7.4	6.6 ± 4.1	<.001	<.001	.02	.26
ΔSBP (mmHg)	1.2 ± 13.5	6.4 ± 8.6	2.4 ± 11.3	5.6 ± 9.6	<.001	.04	.42	.46
ΔDBP (mmHg)	−0.6 ± 7.9	3.5 ± 6.1	0.0 ± 7.0	2.6 ± 6.7	<.001	.008	.53	.26
BMI (kg/m^2^)	22.2 ± 3.3	24.3 ± 3.9	23.2 ± 3.5	23.9 ± 3.0	<.001	.04	.02	.43
Menopause			24.6 ± 4.7	24.9 ± 3.6		.04		
Nonmenopause			22.9 ± 3.2	23.3 ± 4.6		.03		
Smoking history	22.1 ± 3.6	24.2 ± 2.9			.005			
No smoking history	22.5 ± 2.8	24.5 ± 3.1			.003			
Underweight	29 (19.8%)	10 (9.1%)	9 (8.4%)	6 (5.6%)	.02	.41	.01	.32

Abbreviations: BMI, body mass index; HAMA, Hamilton Anxiety Scale; HAMD, Hamilton Depression Scale; HbA1c, glycated hemoglobin; HCY, homocysteine; HDL, high‐density lipoprotein; HY, Hoehn and Yahr Scale; LDL, low‐density lipoprotein; MoCA, Montreal Cognitive Assessment; PD, Parkinson’ disease; TC, total cholesterol; TG, triglyceride; UA, uric acid; UPDRS, Unified Parkinson’s Disease Rating Scale; ΔDBP, orthostatic diastolic blood pressure change; ΔSBP, orthostatic systolic blood pressure change.

^a^ Comparison between male PD and male control.

^b^ Comparison between female PD and female control.

^c^ Comparison between male PD and female PD.

^d^ Comparison between male control and female control.

### 3.2 BMI and sex

Female patients had a higher BMI than male patients (*t* = −2.35, *p* = .02), and the BMI of PD patients of both sexes was significantly lower than that in the respective controls. The BMI levels of female patients and female control with menopause were higher than those of respective nonmenopausal groups (both *p* < .05), and no significant difference was found in BMI between subjects with and without smoking history in either male patients and male control.

In PD patients, multiple regression analysis with BMI as dependent variable and sex as independent variable suggested that the correlation between BMI and sex still remained significant after adjusting for age, duration of disease, HY, UA, HCY values, MoCA, UA, HCY, Δ SBP, Δ DBP, and UPDRS motor scores (*p* = .04).

Furthermore, the prevalence of underweight in male patients (29/146, 19.8%) was two times greater than that in female patients (9/107, 8.4%; *p* = .012). The difference remained significant after we controlled for variables that differed between the two groups (*p* = .016, OR 3.50, 95% CI 1.26–9.75). Further, binary logistic regression analysis indicated that underweight was associated with UPDRS motor scores and lower ΔSBP and ΔDBP values only in male PD (Table 2). In addition, there was no sex difference in the prevalence of underweight (*p* = .32) or correlations between underweight and clinical variables among the controls.

**TABLE 2:** Factors independently associated with underweight by logistic regression analysis in male patients

Variables	*p* Value	Odds ratio	95% CI
ΔSBP	.002	0.92	0.88–0.97
ΔDBP	.003	0.88	0.81–0.96
UPDRS motor	.014	1.09	1.02–1.17

### 3.3 Metabolic factors, BMI, and sex

Linear analyses between metabolic factors and BMI revealed that only the association between UA and BMI was close to significance in male PD group; however, no correlation was observed by multiple regression analysis after adjusting for age and course of disease, and smoking history (*p* > .05), and no correlation was found after adjusting for age, course of disease, and menopause in female patients.

### 3.4 Cognitive function, BMI, and sex

There was no significant difference in MoCA between male and female PD, but male patients were more prominent in language (*p* = .04). MoCA, attention, language, abstraction, visuospatial, and executive function scores of male and female patients were significantly lower than those of controls (all *p* < .05).

Further multiple regression analyses with BMI as dependent variable, and MoCA and subitems of MoCA as independent variables showed that only in male patients, MoCA was correlated with BMI (*p* = .016) after adjusting for age, course of disease and education years, UPDRS motor score, and Δ SBP and Δ DBP values, and there was no correlation between subitems of MoCA and BMI in either group.

### 3.5 Psychiatry status, BMI, and sex

Female patients were more depressed as compared to male patients (*p* = .02). Multiple regression analyses with BMI as dependent variable, and HAMA and HAMD as independent variables revealed an obvious correlation between BMI and HAMD in the female patients (*p* = .042).

### 3.6 Sex difference in associations between BMI and other clinical symptoms

Linear analyses between BMI and all variables found that BMI was correlated with ΔSBP (*r* = .58, *p* < .001) and ΔDBP values (*r* = .41, *p* < .001), HY (*r* = −.37, *p* < .001), HAMA (*r* = −.36, *p* < .001), HAMD (*r* = −.39, *p* < .001), MoCA (*r* = .47, *p* < .001), and UPDRS motor scores (*r* = −.28, *p* = .001) in male patients and BMI was also correlated with ΔSBP (*r* = .45, *p* < .001) and ΔDBP values (*r* = .38, *p* < .001), HY (*r* = −.31, *p* = .001), HAMA (*r* = −.32, *p* = .001), HAMD (*r* = −.45, *p* < .001), and MoCA (*r* = .46, *p* < .001) in female patients after Bonferroni correction (*ɑ* = 0.05/16 = 0.003). As shown in Tables 3 and 4, multiple regression analyses revealed that BMI was independently associated with MoCA, ΔSBP, ΔDBP, and lower UPDRS motor scores in male patients, while BMI was associated with ΔSBP, ΔDBP, and lower HAMD scores in female patients. However, no significant correlations of BMI with clinical symptoms were found in healthy controls of either sex.

**TABLE 3:** Correlations between BMI and clinical variables by multiple regression analysis for male patients

	Unstandardized coefficients		Standardized coefficients	*T*	*p* value	95.0% confidence interval for *B*	
	*B*	*SE*	*β*			Lower bound	Upper bound
(Constant)	20.352	2.479		8.209		15.45	25.254
ΔSBP	0.103	0.016	0.419	6.442	.001	0.072	0.135
MoCA	0.174	0.071	0.235	2.444	.016	0.033	0.314
UPDRS motor	−0.062	0.021	−0.170	−2.865	.005	−0.104	−0.019
ΔDBP	0.077	0.027	0.185	2.838	.005	0.023	0.13

**TABLE 4:** Correlations between BMI and clinical variables by multiple regression analysis for female patients

	Unstandardized coefficients		Standardized coefficients	*T*	*p* value	95.0% confidence interval for *B*	
	*B*	*SE*	*β*			Lower bound	Upper bound
(Constant)	24.814	4.634		5.355		15.612	34.016
ΔSBP	0.075	0.027	0.244	2.812	.006	0.022	0.129
HAMD	−0.116	0.056	−0.244	−2.063	.042	−0.227	−0.004
ΔDBP	0.097	0.045	0.198	2.145	.035	0.007	0.186

## 4 DISCUSSION

To the best of our knowledge, this study is the first to investigate sex differences in underweight and BMI in newly diagnosed and drug‐naïve Chinese patients with PD. The main results of our research are as follows: (a) The BMI levels of both male and female patients were lower than those of the healthy population, and female patients had higher TC, TG, LDL‐C, HAMD, and BMI values and a lower incidence of underweight and HCY levels than male patients; (b) the correlations between underweight and Δ SBP, Δ DBP, and UPDRS motor scores existed only in male patients; and (c) BMI was correlated with ΔSBP and ΔDBP values in both sexes, and BMI was also associated with MoCA and lower UPDRS motor scores in male patients but lower HAMD scores in female patients.

Studies have suggested that significant weight loss can occur in the early stage of PD (Chen et al., 2003; Logroscino et al., 2007), which is consistent with our results; that is, the BMI of both male and female patients was significantly lower than that of the respective healthy populations. The insignificant difference in the prevalence of underweight between female patients and controls may be due to the small number of underweight patients in these two groups, and further study with a larger sample size is necessary.

We found that female patients had higher TC, TG, and LDL‐C levels than male patients, suggesting that women are more likely to develop dyslipidemia. Few studies on sex differences in lipids among PD patients have been reported; however, conclusions similar to ours could be found in the context of other diseases, such as schizophrenia (Li et al., 2016), probably due to the differences in hormone levels and genetics between men and women. Sex differences in smoking rates may lead to differences in HCY levels, as many studies have confirmed that smokers had markedly higher HCY levels than nonsmokers, and HCY levels were related to the daily smoking dose (Iqbal & Yakub, 2013; Panagiotakos et al., 2005). Depression was also more prevalent in female patients, consistent with a previous study (Song et al., 2014).

In this study, we found that female patients had a lower prevalence of underweight and a higher BMI than male patients, and differences in estrogen levels may play an important role. In our study, the average age of female patients was 62 years, indicating that the majority of them were either perimenopausal or postmenopausal. Studies have shown that the reduction in estrogen levels induced by menopause is an independent predictor and trigger of obesity (Davis et al., 2012; Eshtiaghi et al., 2010; Lizcano & Guzmán, 2014). Therefore, although weight loss occurs with the progression of disease, women experience relatively less weight loss than men due to the weight compensation caused by the absence of estrogen. However, Nakamura et al. (2017) reported a higher BMI level in male patients than in female patients. Several possible reasons, such as whether anti‐Parkinson's drugs are taken or not, the duration of disease, the proportion of menopause, physical exercise, dietary pattern differences, and heterogeneity, may lead to different results.

It is interesting to note that underweight was correlated with UPDRS motor scores and lower ΔSBP and ΔDBP values in male patients but not in female patients. The specific reason remains unknown, as no relevant study has been conducted before. We speculated that this may be related to the relatively lower prevalence of underweight in female patients compared with male patients.

Moreover, sex differences exist in the related factors of BMI as well. The close relationships between BMI and Δ SBP and Δ DBP have been well documented in both drug‐naïve and drug‐treated PD patients in recent studies (Nakamura et al., 2017; Umehara et al., 2017), which were concordant with our findings. As retention of salt and water and autonomic activity responses play an important part in the control of orthostatic blood pressure, in patients with lower BMI, the poorer above abilities may lead to lower blood pressure and more significant blood pressure changes (Jordan et al., 1999, 2000; Nakamura et al., 2016).

Studies have suggested that BMI is correlated with cognitive function in treated PD patients (Hughes et al., 2009; Luchsinger et al., 2008); however, the sex difference in this correlation in newly diagnosed patients lacks systematic research. Our results revealed an evident relationship between BMI and cognitive performance only in male patients, although the exact reason is still unclear. Adipose tissue has been reported to be able to produce cognitive‐enhancing substances such as insulin‐like growth factor‐1, N‐methyl‐D‐aspartate (NMDA) receptors and estrogen (Atti et al., 2008; Lorefält et al., 2009); hence, sex differences in body fat and the lower BMI of male patients in this study might partially explain the difference in this correlation.

The association between BMI or decreased body weight and UPDRS motor scores has also been reported in previous studies (Umehara et al., 2017; Wills et al., 2016). In our study, compared with the differences in BMI between female patients and female controls, the differences in male groups were more prominent; therefore, the correlation between BMI and UPDRS motor scores was prone to be positive in male patients.

Interestingly, we found that a negative correlation between BMI and depression severity existed only in female patients, while Pilhatsch's et al. (2013) study showed that the degree of depression was related to reduced BMI in only male PD outpatients. This inconsistency might be attributed to several factors: (a) different assessments for depression—the Beck Depression Inventory questionnaire was used in Pilhatsch's study, and the HAMD was used in our study; (b) different disease severity—patients had more serious motor symptoms in the abovementioned study (average UPDRS motor score was 45), while our subjects were early de novo patients with relatively mild symptoms; (c) concomitant diseases and ethnic heterogeneity can also affect the results. Therefore, sex differences in the correlation between BMI and the degree of depression are worthy of further investigation.

Several limitations should be mentioned in regard to this study. First, the cross‐sectional design prevents the direct reflection of causality between underweight or BMI and clinical symptoms in PD patients. Second, the single‐center data, the inability to perform electromyography for each subject that may miss some people with severe peripheral neuropathy and the absence of other clinical factors, such as thyroid function, all might lead to bias of the results. Third, outpatients were not recruited in the study; therefore, some patients with mild symptoms may have been omitted. Due to a certain misdiagnosis rate of early PD, a long‐term follow‐up is needed to confirm the diagnosis. Fourth, concomitant diseases, lifestyle, dietary patterns, and physical exercise can all have an impact on our findings; therefore, these factors should also be included in future studies.

In conclusion, the present study showed that sex differences existed in BMI values, the prevalence of underweight, demographic, metabolic, and cognitive functions, psychiatry status, and other clinical characteristics in Chinese newly diagnosed and drug‐naïve PD patients. Furthermore, the correlations between underweight and BMI and clinical features also differed between male and female patients. Due to the significant sex differences in early PD patients, sex‐specific management strategies such as dietary structure, physical activity, and nutritional status interventions may have certain clinical significance for improving life quality and prognosis of patients.

## CONFLICT OF INTEREST

There is no conflict of interest to be disclosed.

## AUTHOR CONTRIBUTIONS

Qing Wu, Meizhen Liu and Ming Yu contributed to research design and data collection. Jianfei Fu involved in analyzing data. Qing Wu and Meizhen Liu involved in drafting and revising the manuscript.

### Peer Review

The peer review history for this article is available at https://publons.com/publon/10.1002/brb3.1893.

## Data Availability

The data of this study are available from the corresponding author upon request. The data are not publicly available due to privacy or ethical restrictions.
